# Comparative study of Vertucci’s and Ahmed’s classifications to evaluate the root canal configuration of mandibular incisors in Saudi Arabian subpopulation

**DOI:** 10.12669/pjms.41.6.11972

**Published:** 2025-06

**Authors:** Abdulaziz Sulaiman Alfurayhi, Muhammad Qasim Javed, Safia S. Shaikh, Mansoor Khan, Ayman M. Abulhamael, Syed Rashid Habib

**Affiliations:** 1 Abdulaziz Sulaiman Alfurayhi, BDS Dental Intern, College of Dentistry, Qassim University, Buraidah, Qassim, Saudi Arabia; 2 Muhammad Qasim Javed, FCPS Associate Professor, Department of Conservative Dental Sciences, College of Dentistry, Qassim University, Buraidah, Qassim, Saudi Arabia; 3 Safia S. Shaikh, MDS Associate Professor, Department of Maxillofacial Diagnostic Sciences, College of Dentistry, Qassim University, Buraidah, Qassim, Saudi Arabia; 4 Mansoor Khan Associate Professor, Department of Operative Dentistry, Foundation University College of Dentistry, Foundation University, Rawalpindi 44000, Pakistan; 5 Ayman M. Abulhamael Assistant Professor, Department of Endodontics, Faculty of Dentistry, King Abdulaziz University, Jeddah, Saudi Arabia; 6 Syed Rashid Habib, FCPS Professor, Department of Prosthetic Dental Sciences, College of Dentistry, King Saud University, Riyadh, Saudi Arabia

**Keywords:** Cone Beam Computed Tomography, Dentistry, Endodontics, Mandibular Incisors, Tooth

## Abstract

**Background &Objective:**

The precise anatomical knowledge of root canals can have positive impact on endodontic treatment outcomes. Recently, a new root canal classification system has been proposed by Ahmed. This study aims to compare Vertucci’s and Ahmed’s classification systems to analyze canal configuration of mandibular incisors on CBCT scans.

**Methods:**

The cross-sectional study was conducted in Dental College of Qassim University, Saudi Arabia from May 2023 to April 2024. CBCT 300 images were obtained. Each mandibular incisor tooth was traced axially from the canal orifice to the apex. The canals were classified according to Vertucci’s and Ahmed’s classification systems. Analysis was performed by two calibrated observers. Cohen-Kappa value was calculated to determine the inter and intra observer reliability. The data were analyzed by SPSS-version 26.

**Results:**

CBCT analysis showed that 88.9% of teeth studied were Vertucci type 1 and Ahmed’s ^1^MI^1^. 5.3% fell in Vertucci Type III and Ahmed’s ^1^MI^1-2-1^. 4.4% were of type V Vertucci or Ahmed’s ^1^MI^1-2^. 0.6% teeth could not be classified according to Vertucci classification and were classified as ^1^MI^1-2-1-2-1^. Analysis by Chi-Square test revealed no statistically significant difference in distribution of the canal configuration according to gender or tooth type. The inter-observer and intra-observer reliability values were found to be 0.79 and 0.84, respectively.

**Conclusion:**

The majority of the mandibular incisors of the studied Saudi population were categorized as Vertucci Type-1 and Ahmed’s ^1^MI^1^. Vertucci Type-III and Ahmed’s ^1^MI^1-2-1^ were the most frequently encountered canal types in incisors with two canals. Ahmed’s classification was successful in classifying the whole study sample, whereas Vertucci’s classification was unable to classify five teeth.

## INTRODUCTION

Approximately 15 million endodontic treatments (ETs) are performed each year in the United States, 5 to 10 % of these ETs end up in failure.^1^ In Saudi Arabia the ET failure rate has been reported to be up to 20%.^2^ Common reasons for ET failures include inadequate debridement, missed anatomical features such as additional canals, and insufficient sealing of the canal system. These complications, as well as many iatrogenic errors that compromise treatment success, arise mainly due to an incomplete understanding of root canal morphology and highlight the importance of precise anatomical knowledge in enhancing treatment outcomes.^3^

Many attempts have been made in the past to classify the root canal anatomy and configuration. Vertucci’s system has classically been employed since 1984 to classify the root canal morphology to assist the clinician in gauging the type of root canal configuration (RCC) and plan the ET accordingly. This system was designed after examining teeth via the clearing technique.^4^ Vertucci’s classification presents eight RCCs and does not encompass all variations found in clinical practice. Recently a new classification system has been proposed by Ahmed et al.^5^ This classification system introduces a comprehensive coding method that accommodates a broader range of configurations. The system was developed on micro-CT which is inherently more accurate than all the other traditional methods used for examining and studying the RCC and thus provides a more detailed and precise assessment of the RCC.^5^ The system has also been applied on CBCT scans as Micro-CT is not applicable in vivo.^6^ CBCT is low in cost, has a low radiation exposure, and provides adequate detail for assessing and studying the root canal morphology accurately.^7^

In the majority of cases, mandibular incisors exhibit a single root and a single canal. However, some studies have shown the occurrence of two canals as high as 40%.^8^ A study on a Brazilian population found 20% mandibular incisors with two canals, whereas the frequency of a third canal in the apical and middle thirds of the root was found to be 3% and 1.8% respectively.^9^ This study aims to compare Vertucci’s and Ahmed’s classification systems to analyze canal configuration of mandibular incisors and to categorize those configurations found in mandibular incisors that cannot be classified appropriately using the Vertucci classification system. The study was done on CBCT scans obtained from Saudi Subpopulation of Qassim region. CBCT was preferred as it provides a 3-dimensional detailed observation of the canal morphology in, clinical settings, which contributes to a comprehensive understanding of root canal anatomy.

 The outcomes of this research are expected to enhance clinical decision-making and treatment planning, specifically tailored to the needs of the Saudi population. The study also helps us in measuring the effectiveness of the new classification system in classifying complex root canal systems. This could potentially lead to improved procedural outcomes and patient satisfaction.

## METHODS

The cross-sectional research was carried out at the Dental College of Qassim University, KSA, from May 2023 to April 2024.The sample size (SS) was calculated by utilizing Scalex sample size calculator.^10^ A sample of 364 teeth was calculated as minimum SS with 5% precision, 95% confidence interval, and 61.6% expected prevalence of Vertucci Type-I configuration in Mandibular lateral incisor.^8^

**Fig.1:**
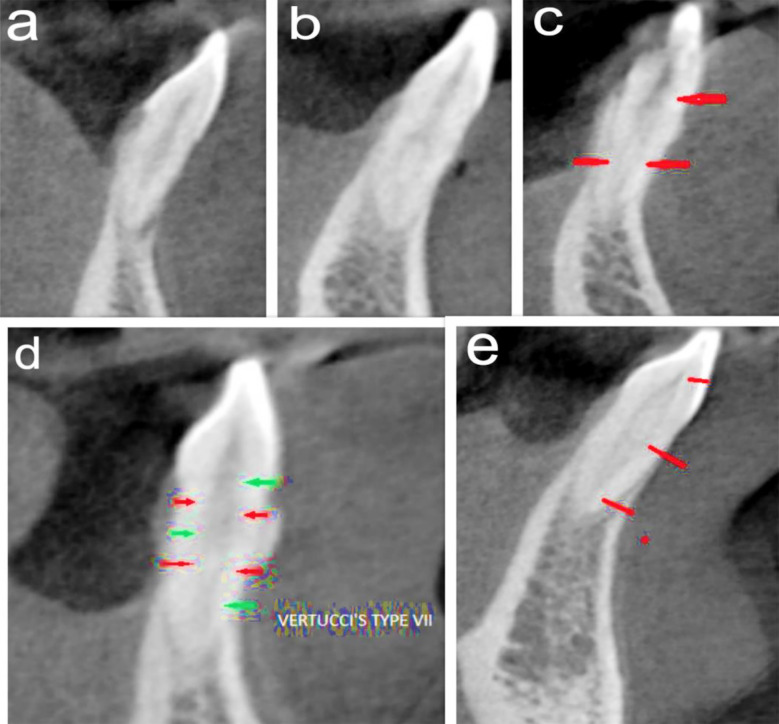
***(a)*** Vertucci’s Type-I, Ahmed’s ^1^MI^1^ ***(b)*** Vertucci’s Type-III, Ahmed’s ^1^MI^1-2-1^ ***(c)*** Vertucci’s Type-V, Ahmed’s ^1^MI^1^-^2^ ***(d)*** Vertucci’s Type-VII, Ahmed’s ^1^MI^1-2-1-2^ ***(e)*** Not classified by Vertucci, Ahmed’s ^1^MI^1-2-1-2-1^.

### Ethical Approval:

It was obtained from the University’s ethical review board (Approval no: 21-16-2; date: April 6, 2022).

A total of 300 CBCT images were obtained from the archives of the Dento-maxillofacial Radiology department at Qassim University Medical City, Dental Clinics.

### Inclusion criteria:

It included CBCT images of the complete mandible of permanent dentition with healthy mandibular central incisors and lateral incisors and healthy periodontium. Any partial or incomplete images, root canal filled mandibular anterior teeth, patients with disease in the mandible including periapical pathology, caries, tumors, cysts, and bony sclerosis were excluded from the research. The mandibular central and lateral incisors that fulfilled the inclusion criteria were included.

### CBCT Imaging and Analysis:

All CBCT images were acquired in high-definition mode with Metal artifact reduction software by utilizing Galileos [Dentsply Sirona; Biensham; Germany] machine. The scan parameters were 3.6 mA, 98 kV, scan time/exposure time of 14 s/2-5 s, field of view (FOV) of 15 cm^3^, and 0.16mm isotropic voxel size. The data reconstruction was carried out in slices at 1mm intervals and analysis was done by Sidexis-4 software. Image analysis was done by two calibrated examiners independently (ASA, SSS). The coronal, sagittal, and axial planes were aligned to horizontal and vertical lines parallel to the long axis of mandibular incisors. Subsequently, analysis was conducted in sagittal and axial sections. Each mandibular incisor (Central and Lateral) was traced axially corono-apically. The RCC was classified according to Vertucci’s and Ahmed’s classification. Two observers underwent calibration session before image evaluation. The six CBCT images with 20 central and lateral incisors were used during calibration and were analyzed twice at an interval of two weeks. The inter-observer and intra-observer reliability was calculated. Subsequently, the aforementioned 20 teeth were excluded from the final data analysis. In the event of disagreement between the two observers, the opinion of a third observer (MQJ) was taken, and cases were discussed until consensus was reached.

### Statistical analysis:

SPSS 26 was used for data analysis. The descriptive statistics were calculated, and chi-square test was used to assess the difference in the distribution of the canal configuration according to gender and tooth type. The Cohen kappa value was calculated to determine the inter-observer and intra-observer reliability.

## RESULTS

The analysis of CBCT showed that out of 802 teeth, 713 teeth (88.9%) were of Vertucci Type-I configuration, which could also be classified as Ahmed’s 1MI^1^ (Table-I and Table-III). 354 (44.1%) of these were mandibular central incisors, while 359 (44.7%) teeth were mandibular lateral incisors. 43 teeth (5.3%) fell in Vertucci class III or 1MI ^1-2-1^ according to Ahmed’s classification, 21(2.6%) of these teeth were mandibular central incisors and 22(2.7%) were mandibular lateral incisors. 36 teeth (4.4%) were of Type-V Vertucci configuration or 1MI^1^-2 as suggested by Ahmed’s classification, with 18 (2.24%) teeth falling in central and lateral incisor groups each. 4 (0.4%) teeth were of Type-VII Vertucci classification or type 1MI^1-2-1-2^ in Ahmed’s classification. 5 (0.6%) teeth could not be classified according to the Vertucci classification and were classified as 1MI^1-2-1-2-1^. Of these, four were central incisors and one was a lateral incisor. None of the teeth exhibited Vertucci Type-IV, VI, and VIII Canal configuration. One hundred ninety two teeth were from female patients, while 610 teeth were from male patients. Analysis by the Chi-square test revealed no statistically significant distribution of the canal configuration according to gender or tooth type (Table-I and Table-II). The inter-observer and intra-observer reliability values were found to be 0.79 and 0.84, respectively.

**Table-I:** Gender-wise distribution and comparison of incidence of Vertucci Subtypes.

		Not classifiable by Vertucci’s Classification	Classifiable by Vertucci’s Classification					P-Value
Gender	N (%)		Type-I	Type-II	Type-III	Type-V	Type-VII	
Male	610 (76.1)	4	538	1	32	31	4	0.576
Female	192 (23.9)	1	175	0	11	5	0	
Total	802 (100)	5	713	1	43	36	4	

**Table-II:** Tooth type wise distribution and comparison of incidence of Vertucci Subtypes.

		Not classifiable by Vertucci’s Classification	Classifiable by Vertucci’s Classification					P-Value
Tooth Type	N (%)		Type-I	Type-II	Type-III	Type-V	Type-VII	
Central Incisors	401 (50)	4	354	0	21	18	4	0.231
Lateral Incisors	401 (50)	1	359	1	22	18	0	
Total	802 (100)	5	713	1	43	36	4	

**Table-III:** Comparison of Ahmed’s and Vertucci’s subtypes.

Ahmed’s	Vertucci’s	N (%)
^1^MI^1-2-1-2-1^	No Vertucci	5 (0.6)
^1^MI^1^	Type-I	713 (88.9)
^1^MI^1-1^	Type-II	1 (0.1)
^1^MI^1-2-1^	Type-III	43 (5.4)
^1^MI^1^-2	Type-V	36 (4.5)
^1^MI^1-2-1-2^	Type-VII	4(0.5)
	Total	802 (100.0)

## DISCUSSION

Results of our study showed that 88.9% of the teeth were of Vertucci Type-I configuration or Ahmed’s 1MI^1^. 5.3% were Vertucci class III or 1MI ^1-2-1^ according to Ahmed’s classification. 4.4% were of Type-V Vertucci configuration or 1MI^1^-2 as suggested by Ahmed’s classification. 0.4% of the teeth were Vertucci Type-VII or type 1MI^1-2-1-2^. Almost 0.6 of the teeth were classified as 1MI^1-2-1-2-1^ and could not be classified according to Vertucci classification. The distribution of canal anatomy did not show any significance according to gender or tooth type.

Vertucci’s classification is the most commonly utilized system for the classification of RCC. However, this classification system is rigid and falls short in identifying different variations in root canal morphologies of several teeth.^5^ Modifications to the Vertucci system have been proposed, which can cater to complex variations in the root canal morphology.^9^ However, many different canal morphologies remain unclassified even when employing the modified Vertucci classification system.^7^,^11^-^16^ This is particularly true in teeth that exhibit high levels of variations, such as the mandibular incisors.

Sevgi et al. reported that 8.34% of the canal system in mandibular incisors in the Norwegian population could not be classified according to the traditional Vertucci classification.^13^ Similarly, De Almeida et al. reported seven different canal configurations in the Brazilian population in mandibular incisors that could not be accommodated by the traditional Vertucci classification system.^14^ Likewise, Leoni et al. and Villa et al. reported 24% and 6.6% mandibular incisors, respectively, which could not be classified according to the Vertucci classification in the Brazilian population.^7^,^15^ Moreover, Wolf et al studied the canal configuration, number of physiological foramen, and frequency of accessory and connecting canals in a German population. He reported four types of configurations that do not fall in the Vertucci classification.^16^

A single root with a single canal may be found in up to 70 percent of mandibular incisors.^3^ In this study, conducted on a Saudi population, the most commonly encountered RCC in these teeth (88.9%) was Vertucci Type-I or 1MI^1^ as described by the Ahmed’s classification. This is slightly on the higher side, especially when comparing the results with the study conducted by Nabil et al who reported Type-I canal configuration to be present in 44.4% of cases^17^. Similarly Taha et al. reported Type-I Vertucci configuration in 76 % of cases in the Jordanian population.^18^ Villa et al. found the Type-I configuration to be present in as little as 50% of the mandibular anterior teeth^7^, while Magat et al. reported the incidence of a Type-I configuration in 73.4% of mandibular anterior teeth in the Turkish population.^19^ Although Type-I may be the most common configuration found in mandibular central incisors, it is prudent for the clinician to closely observe the presence of extra canals, which may harbor etiological factors like bacteria, pulp tissue, or necrotic debris, all of which can lead to post-endodontic treatment failure.^20^,^21^

The second most common configuration found in our sample population was Vertucci Type-III or 1MI^1-2-1^ which was present in 5.3% of teeth. Taha et al.^18^ and Magat et al.^19^ reported Type-III configurations in 18.8% and 20.6% of mandibular anterior teeth. Villa et al.^7^ also reported the Type-III configuration to be present in 20% of mandibular central incisors, similar to the findings reported by Magat^19^, while Nabil et al^17^ reported Type-III configuration in 44.9 % of cases. In this type of configuration, a single canal originates from the pulp chamber and splits into two and then rejoins to exit the root from a single apical foramen. Clinically, if the labial canal is located, prepared, and obturated, which in such cases runs up to the apex, it results in the sealing of the lingual canal and will prevent apical infection.

Vertucci Type-V or 1 MI^1^-2 was the third most common RCC present in about 4.4%. This was slightly lower than Al-Qudah and Awawdah [5.1%]^22^, Mirhosseini et al. (12.9%)^23^ and Da silva et al (14.5% for central and 12% for lateral incisors).^24^ However, our findings were comparable to those reported by Han et al.^25^ (3.89% for central and 5.1% for lateral mandibular incisors). In this type of configuration, as the bifurcating canal is opening into the apical area via a separate foramen, therefore it is essential to locate and treat this canal to prevent post-treatment disease. Extension of the access cavity on the mandibular incisors towards the incisal edge allows better straight-line access and approach to the lingual split from the main canal and increases the ease of negotiation of such canals. This split may occur at any level of the root canal, and the presence of multiple apical exits is also very common in these types of teeth.^26^ Ahmed’s classification is a numbering system with which any variation in root canal morphology in mandibular incisors or any other tooth may be coded. This prevents any morphological variation from being unaccounted for. However, as already mentioned, the Ahmed classification is more of a coding system rather than a classification therefore the accuracy of this coding system in classifying the root canals depends on the sensitivity of the techniques used to map the root canal system. In order to gain accurate information about the root canal system, The clinician, should therefore utilize one or more modalities such as the parallax technique^27^, proper illumination and magnification, and pre-op small FOV CBCT scans.^11^,^28^ A thorough and accurate classification system helps in providing better diagnosis, treatment planning, communication and overall management of endodontic cases.^29^

### Strengths of Study:

This study is the first to compare Vertucci’s and Ahmed’s classification systems using CBCT in mandibular incisors of the Saudi Arabian subpopulation of Qassim Region. CBCT technology provides a more detailed analysis than traditional methods for observing canal morphology, contributing to a comprehensive understanding of root canal anatomy.

### Limitations

The study was conducted only on the Saudi Subpopulation of the Qassim Region with a relatively limited sample size. Therefore, the findings have limited generalizability. Moreover, the CBCT scans used in our study were obtained from machines with relatively large FOV.

## CONCLUSION

Majority of Mandibular incisors of the studied Saudi population were categorized as Vertucci Type-I and Ahmed’s 1MI^1^. Vertucci Type-III and Ahmed’s 1MI^1-2-1^ were the most frequently encountered canal types in incisors with two canals. Ahmed’s classification was successful in classifying the whole study sample, whereas Vertucci’s classification was unable to classify five teeth.

### Future Recommendations:

Future multicenter studies with a large sample size and utilization of CBCT images from small FOV machines will provide greater understanding of the applicability and utilization of Ahmed’s classification for classifying the RCC of mandibular incisors in the clinical settings.

### Grant Support & Financial Disclosures:

The research was funded by Researchers Supporting Project number (RSPD2025R950), King Saud University, Riyadh, Saudi Arabia. The authors appreciate the support from Researchers Supporting Project number (RSPD2025R950), King Saud University, Riyadh, Saudi Arabia.

### Authors’ Contribution:

**MQJ:** Conceived, designed, data analysis, write up, supervised and final approval of the manuscript.

**ASA, SSS**: Data collection, data analysis, Write up.

**MK, AMA, SRH:** Designed, data analysis and Write up.

**MQJ** is responsible and accountable for the accuracy/ integrity of the work.
